# Highly uniform silicon nanopatterning with deep-ultraviolet femtosecond pulses

**DOI:** 10.1515/nanoph-2024-0240

**Published:** 2024-09-05

**Authors:** Eduardo Granados, Miguel Martinez-Calderon, Baptiste Groussin, Jean Philippe Colombier, Ibon Santiago

**Affiliations:** European Organization for Nuclear Research, 162311 CERN, 1211 Geneva, Switzerland; Laboratoire Hubert Curien UMR 5516, Universite Jean Monnet Saint-Etienne, CNRS, IOGS, F-42023, Saint-Etienne, France; CIC nanoGUNE BRTA, Donostia-San Sebastian, Spain

**Keywords:** laser nanostructuring, plasmonics, silicon, accelerators

## Abstract

The prospect of employing nanophotonic methods for controlling photon–electron interactions has ignited substantial interest within the particle accelerator community. Silicon-based integrated dielectric laser acceleration (DLA) has emerged as a viable option by leveraging localized photonic effects to emit, accelerate, and measure electron bunches using exclusively light. Here, using highly regular nanopatterning over large areas while preserving the crystalline structure of silicon is imperative to enhance the efficiency and yield of photon-electron effects. While several established fabrication techniques may be used to produce the required silicon nanostructures, alternative techniques are beneficial to enhance scalability, simplicity and cost-efficiency. In this study, we demonstrate the nano-synthesis of silicon structures over arbitrarily large areas utilizing exclusively deep ultraviolet (DUV) ultrafast laser excitation. This approach delivers highly concentrated electromagnetic energy to the material, thus producing nanostructures with features well beyond the diffraction limit. At the core of our demonstration is the production of silicon laser-induced surface structures with an exceptionally high aspect-ratio -reaching a height of more than 100 nm- for a nanostructure periodicity of 250 nm. This result is attained by exploiting a positive feedback effect on the locally enhanced laser electric field as the surface morphology dynamically emerges, in combination with the material properties at DUV wavelengths. We also observe strong nanopattern hybridization yielding intricate 2D structural features as the onset of amorphization takes place at high laser pulse fluence. This technique offers a simple, yet efficient and attractive approach to produce highly uniform and high aspect ratio silicon nanostructures in the 200–300 nm range.

## 1 Introduction

The rapid progress in nanotechnology has ushered in a new era of innovative materials and devices with unique properties spanning electronics, photonics, and energy storage. Among this extensive library of materials with advantageous properties at the nanoscale, silicon (Si) stands out, owing to its exceptional combination of a high refractive index, minimal absorption losses within the telecommunications spectral range, and its significant technological importance. Silicon nanostructures showcase both electric and magnetic Mie resonances, and provide an effective foundation for achieving unparalleled manipulation of electromagnetic waves on the nanoscale and amplifying light-matter interactions [1], [2]. For example, all-dielectric gradient index metamaterials have been realized for broadband terahertz applications using subwavelength silicon through-hole arrays [3]. At wavelengths relevant for telecommunications, achromatic metasurface lenses have been conceptualized and realized [4]. Silicon nanophotonic devices have also been the focus of intense research towards the next generation of integrated photonic circuits [5], [6].

**Figure 1:**
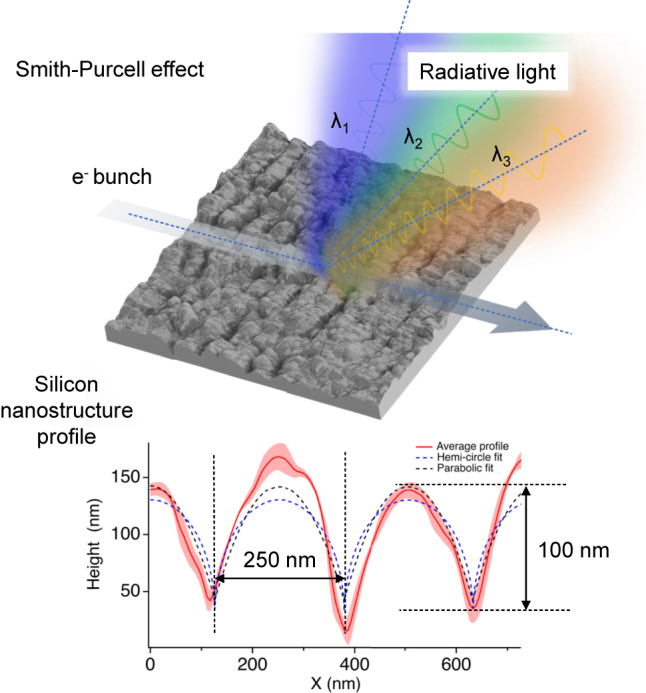
Example of how laser produced nanostructures on silicon can be used for electron spectrometry experiments exploiting the Smith–Purcell effect. This fabrication technique enables nanostructuring with periodicity on the order of 250 nm and substantial aspect ratios and modulation depths exceeding 100 nm.

In recent years, dielectric laser electron acceleration (DLA) has been gaining momentum thanks to its extreme form-factor, low power consumption and low overall cost. Exploiting machine learning methods, a laser-driven silicon nanophotonic electron accelerator was demonstrated [7], and more recently, coherent electron emission and acceleration from a similar device were also observed [8]. Since the radiation generated by the electrons depends on the surrounding matter, nano-engineered materials with tailored electromagnetic and photonic properties provide a unique opportunity to manipulate the interaction between the charged particles and light. Silicon nanostructures, thus, can also be used for non-invasive charged beam characterization and free electron lasing, for example, using the Smith–Purcell effect [9]. The direct correlation between electron energy and scattered light spectrum allows the measurement of the electron beam momentum and energy spread [10]. This emission follows the well-known energy-angle relation *λ* = *L* (*β* ^−1^ − cos *θ*), where *λ* is the radiated wavelength, *L* the period of the structure, *β* = *v*/*c* the normalized velocity *v* of the electron and *θ* the observation angle [11]. Interestingly, even disordered media can be effectively employed for this purpose [12]. Figure 1 shows an example of how laser produced nanostructures can be used for such a task. The key challenge in observing optical Smith–Purcell radiation from a nanograting lies in the alignment of the electron beam with the grating. Cherenkov-based nanophotonics is yet another example where integrated laser-particle interactions may be used for beam diagnostics [13].

In these applications, the relatively affordable requirement for nanostructure quality opens the doorway to a wider range of nanofabrication techniques. Noteworthy, traditional silicon fabrication methodologies, such as chemical vapor deposition (CVD) and lithography, have been extensively harnessed, yet the pursuit of alternative means remains imperative to improve scalability, simplicity, flexibility and cost-efficiency. Emerging at the forefront of innovative fabrication methodologies are laser-based techniques, which have been shown to craft silicon nanostructures with high precision and adaptability over arbitrarily large areas.

Generating sub-wavelength surface periodic nanostructures in high-index semiconductor materials through direct short wavelength femtosecond laser exposure is a well-known technique [14], [15], [16]. This method, also known as laser-induced periodic surface structuring (LIPSS), allows the precise manipulation of nanostructure orientation and morphology by adjusting the polarization direction and illumination conditions. In general, the spatial period (Λ) of the structures obtained via LIPSS is influenced by several factors, including the laser fluence and wavelength (*λ*), the scanning direction, the polarization of the laser electric field, the number of applied laser pulses and their wavefront curvature [14], [17], [18], [19]. In terms of nanostructure spatial coherence over large areas, LIPSS provide an additional advantage thanks to the seeding effect provided by the previously produced LIPSS pattern [20]. Furthermore, even for spatially separated laser scans, it was found that the patterns precisely align thanks to interference effects in the plasmonic coupling between the structures produced by adjacent laser scans [16].

On silicon, low spatial frequency LIPSS (LSFL) has been predominantly observed using femtosecond laser pulse irradiation in air [21]. Generally, LIPSS have been produced using lasers in the near infrared (NIR) [14], [21], [22], [23], [24], [25], [26], [27], [28], [29], [30], [31], [32], [33], [34], [35] and visible spectral ranges [36], [37], however in the deep ultraviolet (DUV) range, only nanosecond pulses at 355 nm [38], 248 nm [39] and 193 nm [40] have been utilized, achieving relatively low aspect ratio nanostructures with maximum modulation depth of up to few tens of nm. Maximizing the attainable aspect ratio of nanostructures by adjusting laser irradiation parameters plays a pivotal role to efficiently exploit nanophotonic effects. While the morphological control and optimization of the attainable LIPSS patterns is limited for constructing a full dielectric accelerator as in reference [7], it is still a useful technique to produce nanostructures for several tasks related to photon-electron coupling in accelerators.

In this context, our study focuses on the surface nanostructuring of silicon through the application of weak DUV ultrafast pulses aided by localized field enhancement. This technique enables nanostructuring on the order of 250 nm and creating structures with substantial aspect ratios and modulation depths exceeding 100 nm. Our approach opens new avenues for tailoring the properties of large silicon areas efficiently. Our approach is interesting as the formation of LIPSS in ultrafast time scales undergoes a considerably different process: the enhanced absorption of radiation, occurring through direct band gap electron excitation of the material, is not able to be transferred to the lattice by heat diffusion.

## 2 Silicon nanopatterning regimes using ultrafast DUV laser pulses

The experiments were conducted in open-air atmosphere with a high repetition rate Yb:KGW laser system. To generate a DUV beam, we employed second and fourth harmonic conversion stages to achieve a final wavelength of 257 nm, which was greatly attenuated to sub-µJ level. The experimental setup is analogous to the one reported in ref. [41] (additional details can be found in the Supplementary Materials). The samples were Czochralski-grown (100) oriented single-crystal N-type silicon, diced and polished to 1,250 µm thick squares of 5 × 5 mm^2^. During the growth process, the samples were chemically combined (doped) with phosphorus gas to make them conductive.

**Figure 2:**
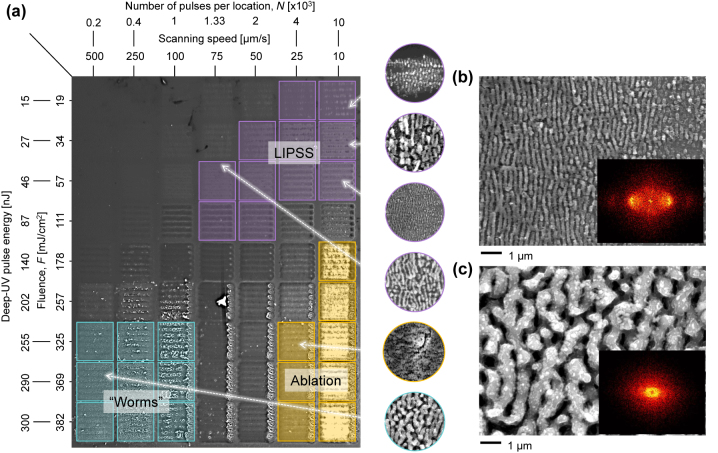
Laser machining parameters (scanning speed, number of pulses por location *N*, deep-UV pulse energy and fluence *F*) at 10 kHz repetition rate for which nanostructures appear. (a) LIPSS are indicated in purple, “worm-like” structures in green and heavily ablated areas in yellow. (inset) Example SEM images at different for different machining setups. SEM images of silicon nanostructures processed with parameters (b) *v* = 50 μm/s and *E* = 46 nJ corresponding to LIPSS, and c) *v* = 500 μm/s and *E* = 290 nJ (“worm-like”). (inset) 2D Fourier transform of the SEM images.

The nanostructure synthesis was performed by scanning the ultrafast laser over the silicon wafer at variable speed but with fixed line inter-spacing of 20 µm over squared areas of 100 × 100 µm^2^ as shown in Figure 2. The repetition rate of the laser was set to *f* = 10 kHz while the DUV beam was focused onto the silicon sample surface with a spot size 2*ω* of 10 µm (1/e^2^ measured using the knife-edge method). We studied different combinations of pulse energy *E* and scanning speed *v*. We group these two variables by sorting the results in terms of cumulative energy deposited per unit area, namely the integrated dose *D* in J/cm^2^, taking into account the fluence *F* multiplied by the effective number of overlapping pulses *N*, and defined as *D* = *F* × *N*.

The resulting nanostructures are shown in Figure 2, where *v* and *E* are modified from left to right and top down, respectively. From this analysis, we see that the parameter space where the nanostructures appear correspond to an integrated dose *D* range of 20–320 J/cm^2^, although *N* and *F* were widely varied in the range from 200 to 10,000 pulses per spot and 20–380 mJ/cm^2^, respectively.

### 2.1 Regular periodic nanopatterning

The parameter space for which high-quality LIPSS structures (i.e. high spatial regularity, coherence and homogeneity) are observed is highlighted in purple in Figure 2a, with a measured Λ of approximately ∼250 nm. The machining parameters were set to a single pulse fluence *F* of approximately 20–100 mJ/cm^2^ with *N* between 1.3 × 10^3^ and 10^4^ pulses per spot. The well-defined LIPSS structures were obtained with an integrated dose *D* in the range of 40–320 J/cm^2^. The LIPSS aligned perpendicular to the laser polarization, which was in turn perpendicular to the scanning direction. When higher pulse fluence *F* was used, the LIPSS appeared along with morphological irregularities arising from direct ablation, reducing the quality of the nanostructures. These are indicated in yellow squares in Figure 2a.

In this regime, the positive feedback amplifies with the effective *N*, ushering in collective effects that induce significant alterations in surface topography. These modifications in individual features, along with their combined interactions, wield considerable influence over both the overall optical response of the system and the periodicity of LIPSS. This concept encompasses three primary aspects: the random distribution and concentration of individual scattering centers, the type of surface nanoroughness, whether resulting from redeposited nanoparticles or nanocavities, and the structural characteristics of the grating itself, under the assumption of pre-established self-organization.

Additionally, it is noted that absorption rates exhibit an increase in a regular grating structure. The localized field enhancement within gradually formed grooves fosters positive feedback as the effective index of the surface approaches unity. This facilitates a smoother transition for incident waves entering the bulk material from air, thereby mitigating reflections attributed to impedance mismatch (as predicted by the Bruggeman effective index model) or to Fresnel reflection effects along the groove sidewalls, ultimately leading to enhanced absorptivity.

The spatial regularity of LIPSS depends on several factors, and the formation process is highly intricate, involving material ablation and relocation that occurs well after the laser pulse has acted. The first few pulses can create random nanocavities capable of seeding the LIPSS formation [42]. For subsequent pulses, it is widely acknowledged that the mechanism behind the LIPSS formation involves the transient excitation of Surface Electromagnetic Waves (SEW). These waves, through interference with the incident laser wave, establish a periodic pattern of laser energy absorption on the irradiated surface, resulting in a modulated temperature distribution [18], [43]. Assuming that the process is mediated by the excitation of surface plasmon polaritons (SPPs), the decay length of SPPs *L*_SPP_ is then linked to the regularity of the periodic structures. The SPP wavenumber *β* for silicon can be calculated following the expression [44]:

(1) $$\beta =\pm \frac{\omega_{l}}{c}\sqrt{\frac{\epsilon_{Si}}{1+\epsilon_{Si}}}$$

where *ω*_*l*_ is the laser center frequency, *c* the speed of light and *ϵ*_*Si*_ the dielectric permittivity of silicon. In general, *β* is a complex number and its real and imaginary part are related to both the mean free path since $L_{\text{SPP}}={\left[2Im\left(\beta \right)\right]}^{-1}$ , and the expected nanostructure periodicity is Λ=2π/Re(β) . The permittivity of silicon is particularly variable in the DUV spectral range [45], most prominently around 4.9 eV. For instance, for our excitation wavelength at 257 nm (4.82 eV), we retrieved an interpolated value of *ϵ*_*Si*_ = −12.77 + *i*12.95, corresponding to *L*_SPP_ ≈ 1 µm and Λ ≈ 250 nm. These results are in good agreement with the usually obtained *L*_SPP_ of the order of a few microns when using longer irradiation wavelengths for highly regular nanostructuring [43]. To illustrate this, we have performed 2D FFT calculations of the scanning electron microscope (SEM) images of the produced nanostructures shown in Figure 2b. A way of quantifying the spatial regularity of the produced nanostructures is by calculating the dispersion in the LIPSS orientation angle (*δθ*) (also known as DLOA). Generally, surface patterns with few bifurcation points and high spatial coherence yield DLOA angles smaller than 15°, while patterns with a low degree of spatial coherence yield a DLOA of up to 50° and beyond [43]. The calculation performed using 2D-FFT of our SEM images results in a value of *δθ* ≈ 10°, indicating a high degree of regularity.

**Figure 3:**
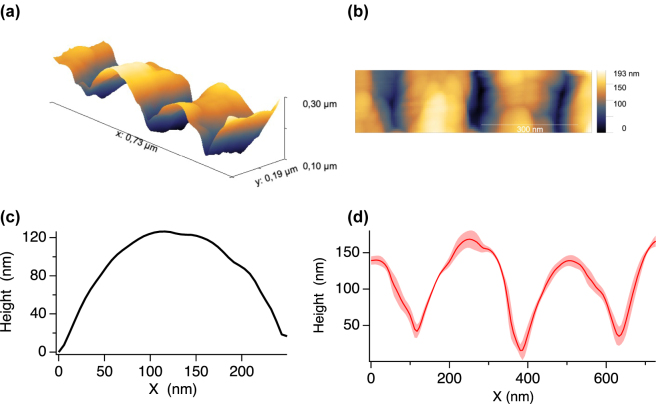
Topography of laser produced nanostructures. (a) 3D visualization of the topography of LIPSS nanostructures created at low fluence and low scanning speed as observed by AFM. (b) Representative AFM micrograph. (c) Averaged height profile and (d) averaged profiles of the resulting grating-like nanostructures.

To study the surface topography in more depth, we conducted atomic force microscopy (AFM) measurements. A “JPK NanoWizard V” produced by Bruker was used for the surface roughness investigation. The AFM nano-tip used was a SCOUT 150 high aspect ratio silicon probe (Nu Nano Ltd) with a cone angle of < 15° over the final 1 µm of the 5 nm radius tip ( < 10 nm). The AFM images depicted in Figure 3a and b illustrate the three-dimensional surface details of LIPSS created using optimized number of pulses for producing LIPSS with the largest aspect ratio (pulse energy of 46 nJ and speed of 50 μm/s). As it can be seen in Figure 3b, the maximum peak to valley value attained is 193 nm. Figure 3c shows an averaged profile demonstrating more than 120 nm modulation and a hemi-cylindrical type shape. Figure 3d depicts statistics of profiles of consecutive grooves. A large area study of the nanostructures’ heights revealed an average LIPSS height ranging from 100 to 120 nm, which increased with higher effective pulse number *N*.

### 2.2 Surface pattern hybridization at high laser pulse fluence

A different type of structures, resembling worms with size approaching 1 µm, were observed with single pulse fluences *F* above 300 mJ/cm^2^, with a reduced number of pulses *N* (scan speed was set between 100 and 500 μm/s, corresponding to *N* from 200 to 1,000 pulses and an integrated dose *D* range of 65–380 J/cm^2^). The parameters for which these worm-like structures appeared is indicated in blue in Figure 2a. Worm-like structures have received relatively scant attention in the literature, emerging as a recent topic that addresses the intricacies of anisotropic structures with a two-dimensional density, diverging from the conventional one-dimensional perspective. Speculatively, within this ablative regime, nanoparticles are likely generated and redeposited by preceding pulses. Their size is presumably contingent upon the thickness of the expelled liquid layer, barring any plasma recondensation. The optical coupling of subsequent pulses with these nanoparticles could potentially induce collective Mie resonances, akin to those characterized in Si nanoparticle arrays, as reported in a recent article [46]. Additionally, mode hybridization may give rise to surface lattice resonances (SLR), promoting structures parallel to the incident polarization around each protruding nanorelief [47].

**Figure 4:**
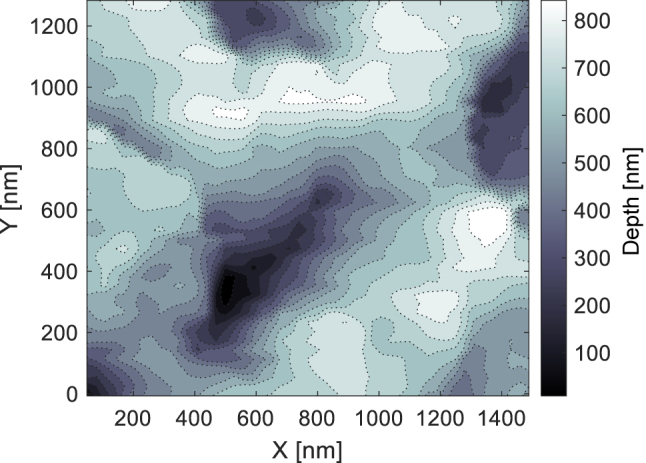
Surface topography of “worm-like” nanostructures as observed by atomic force microscopy.

Previous observations [48] have indicated that bump-type structures on metals favor near-field coupling parallel to the electric field (E). If surface plasmon polaritons (SPPs) perpendicular to $\vec{E}$ on silicon with metallic properties in the UV spectrum and SLRs capable of generating parallel structures, the random deposition of nanoparticles could give rise to numerous imperfections, thereby shaping a network following two dimensions, replete with myriad bifurcations reminiscent of worm-like structures. The nanostructures, in this case, present features of around 1 µm and small protuberances of around 100 nm. The 2D FFT reveals an annular shape characteristic of homogeneous radial distribution of nanostructure features, as shown in Figure 2c. The origin of these worm-like structures is currently unknown. However, given that the fluence is close to the damage threshold of silicon [49], we suggest that this formation mechanism may be linked to an ablative or melting process. The “worm-like” AFM measurements revealed no apparent periodicity but large peak-to-valley depth values of more than 800 nm, as shown in Figure 4.

### 2.3 The onset of amorphization

**Figure 5:**
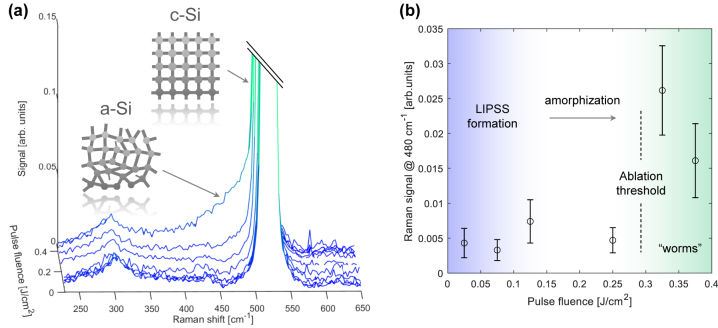
Laser treated surface chemical characterization. (a) Raman spectra of the laser processed silicon samples for single laser pulse fluence ranging from 0 mJ/cm^2^ (unmodified surface) to 380 mJ/cm^2^. A prominent shoulder in the 400–500 cm^−1^ range appears for “worm-like” nanostructures produced at maximum fluence. (inset) Raman peaks of the silicon sample for crystalline (c-Si) to amorphous (a-Si) phases. (b) Raman signal at 480 cm^−1^ as a function of single pulse fluence. Transition from LIPSS to “worms” nanostructuring regime and its comparison to the silicon ablation threshold.

Raman spectroscopy was employed to assess whether the femtosecond laser treatment had any impact on the overall physical and surface chemistry characteristics of the Si substrate. The spectral data was collected at room temperature using a Nd:YVO_4_ laser operating at 532 nm (Oxxius), utilizing a back-scattering geometry. This was accomplished using a confocal Raman microscope Alpha 300R WiTec with a spectral resolution of approximately 1 cm^−1^, featuring a cryo-cooled charge-coupled device (CCD) detector. The laser spot size was approximately 2 µm. Figure 5a displays the spectra obtained from the various silicon samples as a function of laser pulse fluence. The Raman peaks in crystalline semiconductors exhibit sharpness due to the precise wave vectors associated with the phonons. The first-order Raman (Stokes) spectrum features a prominent peak at 520.8 cm^−1^, which originates from the generation of the triply degenerate, long-wavelength transverse optical phonon (TO) in the silicon lattice.

Our primary focus was on the Raman spectra within the spectral range of 400–550 cm^−1^. The characteristics of this peak are defined by its position, with a full width at half maximum (FWHM) of approximately 3.5 cm^−1^ at room temperature [50], as well as its intensity. The shape of this scattering band conveys information about the deformation of the silicon crystal lattice, making it susceptible to alterations caused by the laser nanostructuring process. In fact, a broader c-Si Raman peak at 580 cm^−1^ is a signature of polycrystalline silicon.

In the case of LIPSS nanopatterns, the Raman analysis shows that the overall physical and surface chemistry remained unchanged from that of untreated Si, highlighting the effectiveness of the ultrafast DUV laser technique for precise nanopatterning without altering material properties. However, for “worm-like” silicon nanostructured samples, a visible shoulder appeared in the Raman signal at around 480 cm^−1^ compared to the LIPSS and pristine surfaces. The downshift of silicon peaks to lower wavenumbers suggests the presence of lattice stress in the treated areas, although such a downshift could also be related to a size effect of the amorphous structure of the Si shell (characteristic *α*-Si band). Although a full transition to amorphous silicon was not evident, the widened spectra indicated the existence of an amorphous layer on the surface. This is shown in Figure 5b, where the Raman signal in this band is plotted as a function of laser pulse fluence. This suggests the creation of defects during the LIPSS generation process. In all nanostructured areas, the enhanced absorption at visible wavelengths was evident when comparing the processed and pristine areas.

## 3 Digging deeper: positive feedback and field enhancement effect

Typically the modulation depths of LIPSS nanostructures approximately match the optical penetration depth at the wavelength used within the laser-excited material [21], [51]. Modulation depths of 100–200 nm were achieved for NIR illumination, but only 10 nm was demonstrated in the DUV range [38], [39], [40]. Interestingly, in this work, the observed modulation depth exceeded 100 nm. The measured high aspect ratios can be of great interest for nanophotonic applications and the understanding of this finding is crucial.

During the LIPSS formation process, the structures created in the first few laser pulses have a preferred orientation perpendicular to the polarization of the driving field. Subsequently, a faint yet discernible well-aligned Si-nanostructure usually appears for a low number of impinging pulses *N*. It is worth noting that a few initial pulses are necessary to create a sufficiently rugged surface, initiating the pattern formation. The depth of these structures is related to the laser energy absorbed at the surface, and is localized within a layer determined by the depth of light penetration. The Beer–Lambert law provides a useful method for measuring the depth at which light can penetrate. According to this law, light intensity diminishes exponentially as it progresses through a material depth [52]. Following this rationale, the modulation depth obtainable should be inversely proportional to the effective attenuation depth of the material. The attenuation of silicon at 257 nm (4.82 eV) is *α* = 1.9 × 10^6^ cm^−1^ compared to 0.78 × 10^3^ cm^−1^ at 1.5 eV [45]. This logic contradicts our experimental results, since we obtained groove depths similar to those with illumination at NIR wavelenghts.

A possible explanation for this large modulation depth comes from the field enhancement around these early nano-patterns produced by the first laser pulses impinging the surface [42]. Here we assumed that the process is initiated by SPP, however, the possible presence of evanescent cylindrical waves (CW) cannot be disregarded [53], [54]. It has been shown that the minimum possible periodicity (Λ_min_) for silicon LSFL is mainly influenced by the excited electron density *N*_*e*_. The evolution of the surface morphology imposes here the dominant contribution to the pattern from both SPP and CW [18]. The multipulse effect is complicated because of the inhomogeneous distribution of free carriers in the crests and valleys once the nanostructures are formed. Consequently, light absorption is enhanced due to the nanogroove morphology during the growth process [53].

**Figure 6:**
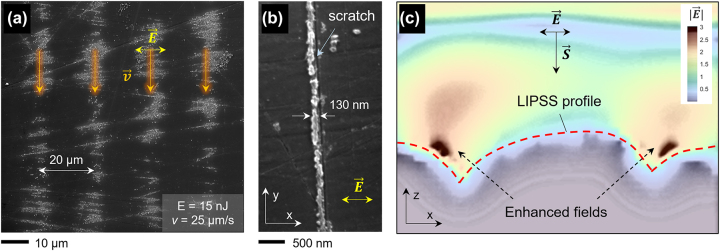
Nanopattern formation seeded by pre-existing surface morphology. (a) SEM image of the processed silicon sample at the fluence threshold for LIPSS formation (pulse energy of 15 nJ and processing speed of 25 μm/s). $\vec{E}$ indicates the laser polarization and $\vec{v}$ the sample displacement direction. (b) Evidence of LIPSS seeding: a single 130 nm wide nanogroove is produced (corresponding to *λ*/2) in the vicinity of a nano-scratch. *x* and *y* are the coordinates contained in the silicon surface plane. (c) FDTD simulations result of the absolute value of the electric field $\left|\vec{E}\right|$ in the vicinity of the produced nanogrooves under 257 nm light illumination. *z* coordinate is perpendicular to the silicon surface and parallel to the Poynting vector $\vec{S}$ . The input profile for the simulation is taken from the AFM measurements.

The positive feedback induced by laser-induced nanoroughness or previous surface defects can be clearly observed in processed areas near the threshold for LIPSS formation (Figure 6a). Here, the structures appear predominantly around nano-scratches obtained during the polishing of the sample. Furthermore, Figure 6b shows a single nano-groove of 130 nm width produced in the vicinity of a scratch, which had a length of over 30 µm. In fact, it has been recently demonstrated that controlled artificial nano-features (seeds) can serve as a seeding mechanism for LSFL nanopatterning with improved spatial regularity [20], [55].

To shed light on this problem, we performed 3D finite-difference time-domain (FDTD) simulations using the AFM measurements of the produced LIPSS patterns illuminated with 257 nm, resembling the fields during the production stage. Figure 6c shows the calculated absolute value of the electric field distribution in the region close to one of the grooves when illuminated at normal incidence with light at 257 nm polarized perpendicularly to the grooves. The simulation result shows that the laser fields are enhanced significantly (by at least a factor of 3) in the vicinity of the grooves’ deepest point. We suggest that the dynamic morphology appearing at the surface of the silicon during the multi-pulse LIPSS-ablation process acts as a reinforcement, facilitating the increase of the pattern aspect ratio (or groove depth) as the number of pulses *N* is increased. It is worth noting that even though the single pulse fluence was well below 100 mJ/cm^2^ in all cases, taking into account the field enhancement factor, the peak fluence can easily reach the silicon ablation threshold at sub-ps timescales of around 0.2–0.5 J/cm^2^ [14].

Generally, multi-pulse changes in the surface topography are driven by the interplay between electromagnetic and hydrodynamic effects. Through the profile deepening and roughness amplification mechanism, LIPSS are not erased but become more pronounced by electromagnetic effects and pulse-by-pulse material removal. The local absorbed electromagnetic energy induces a strong localization of the electron temperature, which, after the relaxation, induces a spatially inhomogeneous heating and phase transitions. After *N* pulses the energy distribution is assumed to follow the distribution of the resulting interference field with the hydrodynamic response of the material. In our simulations, the field enhancement effect observed during the groove formation stage indicates that the feedback effect is limited to a depth of approximately 80 nm. It is expected that the pulse ablation rate varies non-linearly following the trend of the absorbed energy in the vicinity of the surface. This nonlinear increase in the profile height for the first pulses is due to positive feedback, especially in the depression regions. For a higher number of pulses (likely estimated at *N* > 200), saturation is observed as the crests absorb more and more energy, as much as the valleys, leading to the ablation of the peaks. In this regime, a form of regulation occurs, and although the ablation per pulse within the spot increases, the LIPSS profile remains practically stationary with increasing *N*. This phenomenon has been reported and explained, for instance, in ref. [56]. However, a detailed study of the correlation between groove depth and number of impinging pulses *N* is necessary to clarify this matter.

**Figure 7:**
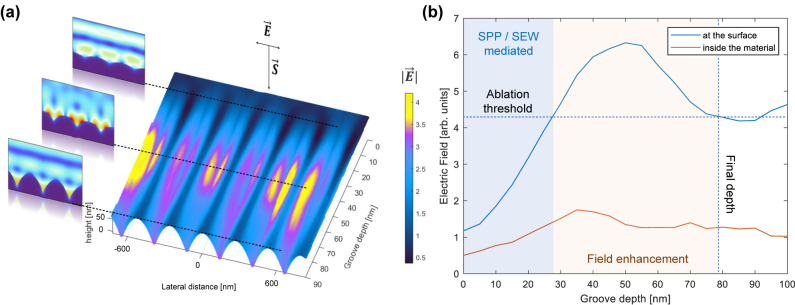
Simulations of the laser electric field at the silicon surface during the production stage. (a) Field enhancement simulation for ideal parabolic nanostructures as a function of groove depth. (b) Regions of nanostructuring due to the excitation of surface plasmons and electromagnetic waves, field enhancement, and final nanostructure aspect ratio.

Figure 7a shows the computed laser electric field on the surface of a nanopattern varying its depth from 0 to 90 nm. A maximum field enhancement of a factor of 4 is observed. Figure 7b shows the peak of the electric field as a function of the groove depth, indicating that the field is maximal for grooves of 40–50 nm depth, and decays until the depth of 80 nm is reached corresponding to the ablation threshold. When the enhanced laser field equals the ablation threshold the final depth is reached (additional FDTD simulations can be found in the Supplementary Materials). In contrast to LIPSS formed in metals, LFSL in silicon are usually obtained with fluence conditions below the melting point. This submelting condition has already been reported by other authors [57]. Our Raman spectroscopy experiments showed minimal presence of an amorphous phase for LIPSS formation, suggesting also that a liquid layer was not formed during irradiation.

## 4 Conclusions

In conclusion, we have demonstrated the formation of silicon LIPSS patterns with 250 nm periodicity and more than 100 nm height using an ultrafast DUV laser setup employing very weak pulses. The structures orientation was found to be solely dependent on the laser polarization with periodicity close to the laser wavelength. Amorphization of the surface occurred for single pulse fluences higher than 0.2 J/cm^2^ during the process. The patterning process may be extended to virtually unlimited surface areas and the low requirement for pulse energy ensures high processing speeds. The LIPSS morphology consists of ridges varying in height (100–150 nm) depending on the effective pulse number *N* for a range of pulse fluence between 50 and 150 mJ/cm^2^. The obtained aspect ratio represents an increment of around an order of magnitude when compared to previous works utilizing comparable irradiation wavelengths but at longer pulse duration. We suggest that the mechanism behind the formation of high aspect ratio LIPSS relies on field enhancement effects during the multi-pulse irradiation regime.

At higher laser pulse fluence, we obtained intricate 2D patterns arising possibly from mode hybridization and subsequent surface lattice resonances -which promote parallel structures to the incident electromagnetic field instead. Presumably the optical coupling of subsequent pulses with redeposited nanoparticles could potentially induce collective Mie resonances, although more work is needed to shine light onto this hypothesis.

To sum up, this work demonstrates the synthesis of Si nanostructures without relying on chemical methods. The shown method guarantees the purity of the fabricated nanostructures and offers customization options. We envisage that the developments highlighted in this study will pave the way for applications, particularly in silicon light-based electron detectors and integrated dielectric laser acceleration.

## Supplementary Material

Supplementary Material Details
