# Supplementary material for: Highly uniform silicon nanopatterning with deep-ultraviolet femtosecond pulses
Source: Nanophotonics. 2024 Sep 5;13(22):4079–89. doi: 10.1515/nanoph-2024-0240 (PMC11501060; doi:10.1515/nanoph-2024-0240)
Supplement: Supplementary file 1 — Supplementary Material Details [file j_nanoph-2024-0240_suppl_001.pdf]

Supporting information: Highly uniform silicon nanopatterning with deep-ultraviolet femtosecond pulses

E. Granados[1]*, M. Martinez-Calderon[1], B. Groussin[1], J. P. Colombier[2], and I. Santiago[3]**

[1]CERN, European Organization for Nuclear Research, 1211 Geneva, Switzerland
[2]Universite Jean Monnet Saint-Etienne, CNRS, IOGS, Laboratoire Hubert Curien UMR 5516, F-42023, Saint-Etienne, France
[3]CIC nanoGUNE BRTA, Donostia-San Sebastian, Spain

email: * eduardo.granados@cern.ch, **i.santiago@nanogune.eu

In this document, we provide additional information and data related to the fabrication of the nanostructures on silicon, as well as the details of the experimental setup. The results are accompanied by COMSOL simulations of field enhancement achievable with modeled silicon laser induced surface structures (LIPSS).

Contents

1 Previous works of LIPSS on silicon **2**

2 Experimental setup **3**

3 Additional FDTD and electromagnetic simulations **4**

4 Additional data of surface analysis using SEM and AFM **6**

1 Previous works of LIPSS on silicon

Femtosecond laser processing of Si was pioneered by Mazur's group more than 25 years ago [1]. In this seminal work, the authors succeeded in producing high aspect ratio silicon microstructures with the aid of chemical reactions involving SF_6 and Cl_2. The technique was also utilized with various pulse durations and wavelengths to produce pillar-like silicon nanostructures [2, 3]. Recently, the direct gas-free laser fabrication of micro- and nanostructures in semiconductor materials has attracted keen interest in the scientific community. This has prompted extensive works demonstrating deep sub-wavelength silicon nanostructuring using ultrafast laser excitation in air [4–7]. Silicon nanostructuring was also employed for producing highly absorptive "black silicon" [8], generating significant interest in the solar photovoltaic sector. This is due to its ability to enhance the light-to-electricity conversion efficiency of conventional crystalline silicon solar cells [9–13].

The first direct-laser processing investigations in Si were carried out under nanosecond pulse excitation in the visible and near-infrared (NIR) spectral regions [14]. With the advent of ultrafast lasers in the 90s, the focus quickly shifted towards NIR femtosecond laser excitation. Ultrashort laser beams can efficiently initiate non-linear processes like multiphoton absorption and tunneling ionization, which involve virtual states. These effects collectively facilitate the excitation of electrons from the valence band to the conduction band through the multiple absorptions of sub-bandgap photons. In fact the majority of studies carried out in silicon so far have been mostly in a range of NIR wavelengths (800 - 1030 nm) [15–30] and a few in the visible range at 400 and 515 nm [31, 32] or combinations of them [33–35]. A non-exhaustive summary of these previous works is compiled in Figure S1.

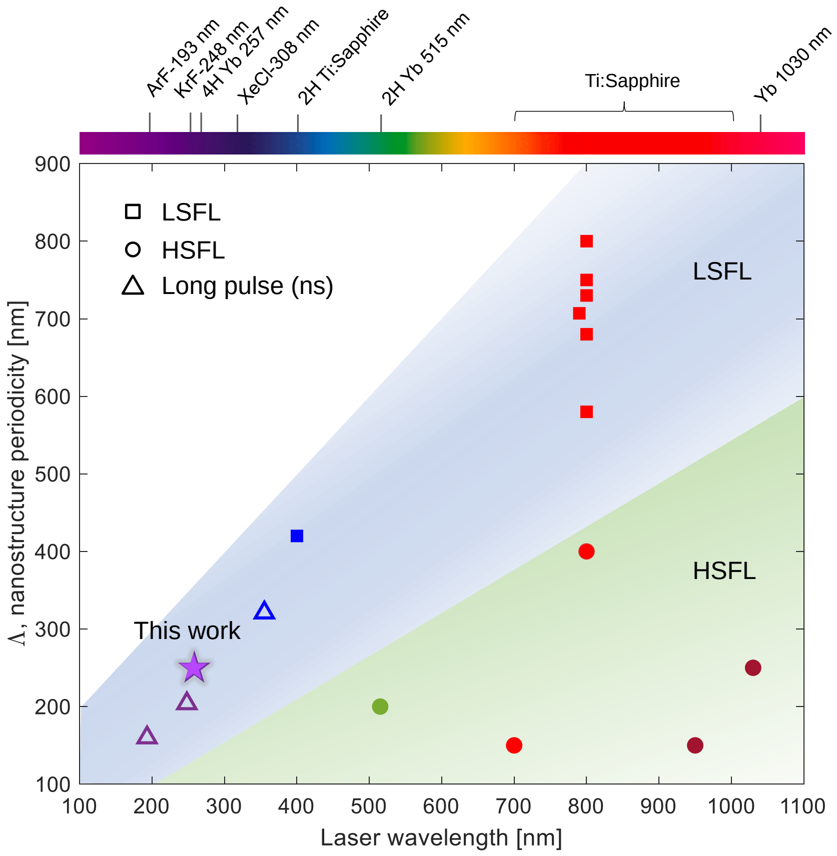

Figure S1: Non-exhaustive compilation of the obtained silicon nanostructure periodicity (Λ) using laser induce periodic surface structuring (LIPSS) as a function of laser technology and irradiation wavelength. In general, two regimes can be found: High spatial frequency LIPSS (HSFL) and low spatial frequency LIPSS (LSFL) depending on the attained periodicity of the nanostructures. This work presents the results obtained using high repetition rate ultrafast deep ultraviolet (DUV) excitation.

For LSFL, Λ is associated with the interaction between the incident laser beam and surface plasmons or surface plasmon polaritons (SPPs). This phenomenon occurs due to the weak in-plane interference

of the incident transverse laser pulse and nearly transverse surface polaritons. These nanostructures are predominantly observed near the ablation threshold, resulting in a significant periodic modulation of the surface topography. Furthermore, silicon wafers used in common LIPSS fabrication studies are considered inert, but this assumption needs to be revisited as, in some cases, LSFL orientation cannot be explained using the current LIPSS theory [36]. The exact mechanisms underlying the formation of HSFL remain not fully understood either [37].

Although it is known that there is a correlation between the ripple period and laser wavelength, there is a scarcity of research focused on the generation of LIPSS caused by DUV lasers. The majority of the works in this spectral range have used nanosecond pulses at 355 nm [38], 248 nm [39] and 193 nm [40]. The limited research in this wavelength range could be attributed to the prevalence of non-polarized light emission from most available DUV lasers, which poses a barrier to the formation of LIPSS [40]. Moreover the attained LIPSS structures had modulation depth of around 10 nm or less. To the best of our knowledge femtosecond DUV excitation has remained unexplored to date. This parameter space is interesting as the formation of LIPSS in ultrafast time-scales undergoes a considerably different process: the absorption of radiation, occurring through direct band gap electron excitations of the material, is not able to be transferred to the lattice by heat diffusion.

2 Experimental setup

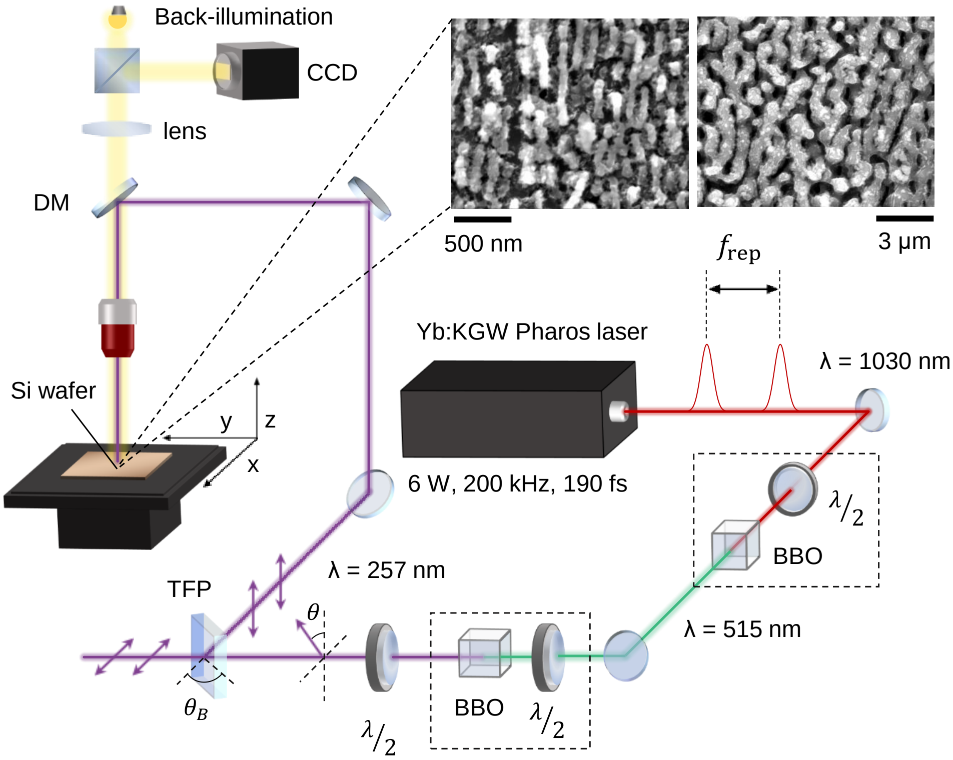

Figure S2: Experimental optical setup featuring an IR femtosecond laser, its harmonic conversion to DUV, the nanopositioning stage for sample handling and the imaging system. DM: Dichroic Mirror, TFP: Thin-film polarizer, BBO: Beta Barium Borate, Θ_B: Brewster's angle (inset) Example images of the nanostructured silicon wafer as seen by SEM.

We used a Yb:KGW Pharos laser from Light Conversion for our silicon surface processing setup, which is depicted in Figure S2. The laser was capable of delivering up to 6 W average power at 1030 nm, with a pulse duration between 190 fs and 10 ps at up to 200 kHz repetition rate. In order to produce a DUV beam, second and fourth harmonic conversion stages were used based on Beta Barium Borate (BBO) crystals to reach a final wavelength of 257 nm. We precisely adjust the DUV energy in a range between 10 nJ and 300 nJ by rotating a motorized $\lambda/2$ waveplate to tune the amount of S-polarized light transmitted through a Thin Film Polarizer (TFP). The average power deposited on the sample did not

exceed 10 mW in any of the experiments performed. The S-polarized beam is then steered towards a UV femtosecond-grade 100 mm focal length lens and focused on to the silicon sample surface with a spot size 2ω of 10 µm ($1/e^2$). Patterning the surface with sub-micron accuracy was achieved by moving the sample using a 3-axis motorized stage with down to 200 nm repeatability. Finally, a microscope imaging system placed on top of the machining line was used to have live visual feedback over the machining operations at the few-micron scale.

The UV beam energy delivered to the sample was online monitored during all the tests by sampling a portion of the beam before the scanning translation stages and regulated using the motorized waveplate. The UV beam was introduced into the machining setup through a dichroic mirror, which allowed to simultaneously image the processed sample live.

The S-polarized beam was then directed towards a UV femtosecond-grade lens with a focal length of 100 mm and focused onto the silicon sample surface, producing a spot diameter (2ω) of 10 µm ($1/e^2$). Achieving sub-micron precision in surface patterning was possible by manipulating the sample position using a 3-axis motorized stage with repeatability down to 200 nm. To monitor and ensure precise machining operations at the sub-micron scale, we employed a microscope imaging system situated above the machining setup, providing real-time visual feedback during the processing.

3 Additional FDTD and electromagnetic simulations

The objective of the simulations is to examine the response of three-dimensional silicon morphologies present in the experimentally tested samples when exposed to a DUV laser beam perpendicular to them. By roughly tailoring the surface structures in size and shape, it is possible to induce the excitation of localized field enhancement.

The AFM data shown in the main text clearly revealed the presence of periodic ripples known as laser induced periodical surface structures (LIPSS) [37], with periodicities ranging from approximately 250 nm to 260 nm. In our modelling approach, we construct parabolic nanostructures that are periodically placed on a surface. The aspect ratio was established from the measurements based on AFM data so that the height is approximately 100 nm. To maximize the field enhancement factor, the structures were oriented perpendicular to the polarization of the incident wave.

Figure S3a and b illustrate the transmission outcomes from the FDTD simulation. A notable contrast is evident in the transmitted fields penetrating the silicon substrate when comparing a flat surface to a nanostructured one. In accordance with the absorption coefficient behavior of silicon, photons with energies exceeding 2.5 eV (corresponding to wavelengths shorter than 500 nm) are entirely absorbed within the initial tens to hundreds of nanometers. For wavelengths spanning from 500 to 800 nm, Figure S3b shows that the LIPSS nanostructures diffract incoming light, leading to periodic constructive interference at varying depths. As anticipated, these depths change with the wavelength, generating fields at least four times more significant than those achieved with a flat silicon surface.

In our reflectivity simulations, we started by considering the geometry of the nanostructures. Various surface configurations display different profiles of gradient refractive indices, including linear, parabolic, cubic, exponential, or sinusoidal forms, influencing transparency and bandwidth of the textured surface. Particularly in this study we assessed the morphology and measurements of these structures using AFM traces. Our analysis indicates that the average morphology falls somewhere between hemicylindrical and triangular shapes. However, we sought to compare the modeled nanostructure's response with that of the AFM-derived ones. We used the simulation software COMSOL to solve Maxwell's equation in a 3D domain with its embedded Finite Element Method (FEM) solver for differential equations and calculated the reflectivity at each wavelength parametrically.

The graph in Figure S3c presents the computed reflected spectrum for various models of LIPSS, alongside the outcomes derived from the FDTD simulation employing the AFM data. Notably, the actual behavior of LIPSS appears to lie between that of ideal triangular and hemicylindrical LIPSS for wavelengths larger than 400 nm. Among the shapes investigated, perfectly formed hemicylinders display the most favorable performance, demonstrating minima around 300 nm with only a few percent reflectivity. Across the wavelengths between 500 and 1000 nm, the theoretical reflectivity of the modeled LIPSS ranges between 20% and up to 30% throughout the entire spectral range. It is essential to highlight that these simulations do not factor in the impact of amorphous phases in the treated silicon, thus representing an upper limit for the reflectivity.

We performed additional electromagnetic simulations using COMSOL™ to determine the expected value of the peak field for a parabolic nanostructure resembling the ones obtained experimental as a function of impinging wavelength. We employ the Finite Element Method (FEM) solver to solve Maxwell's

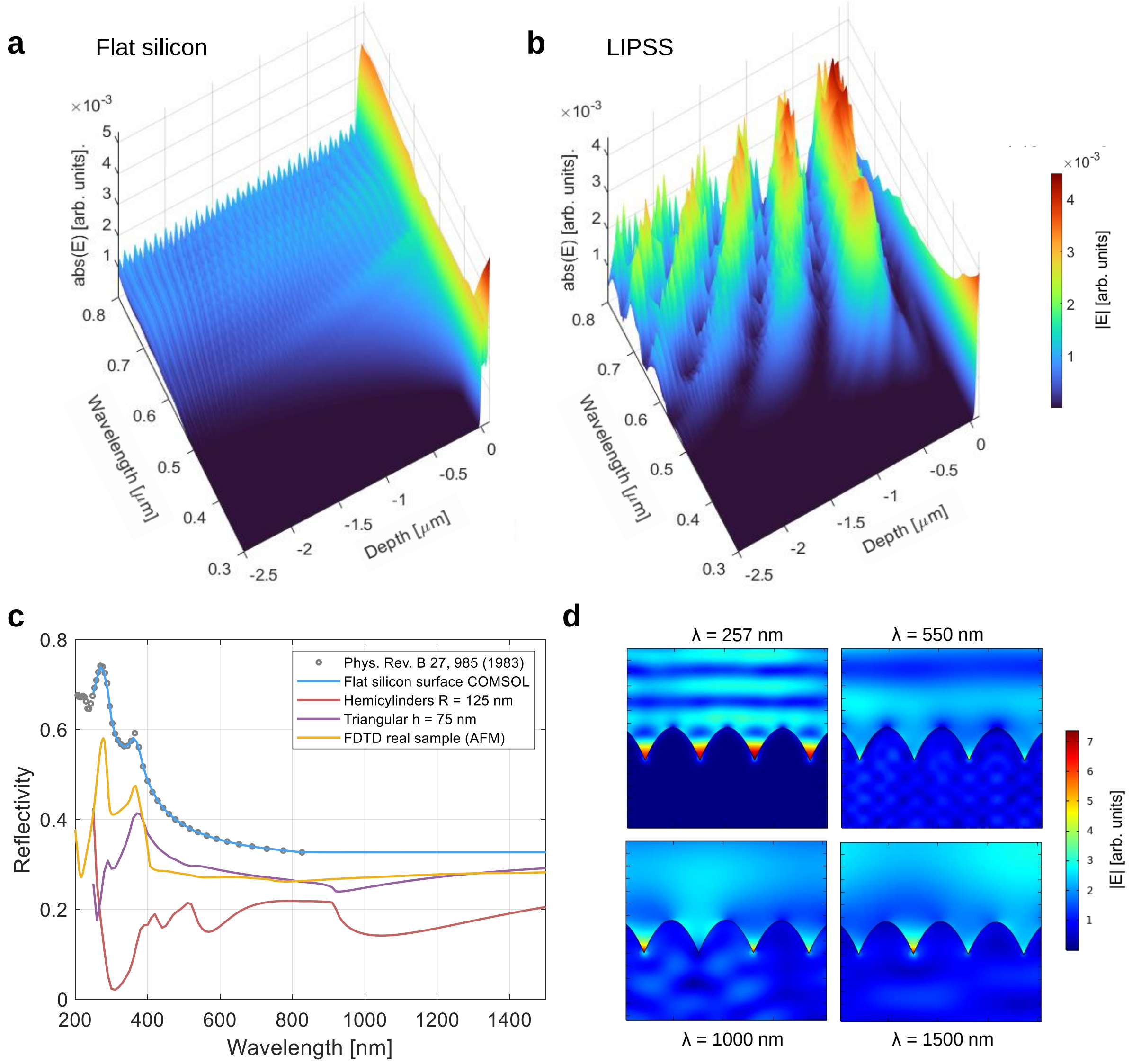

Figure S3: FDTD simulation results of the transmitted electric fields for a light source between 300 and 800 nm. Results are shown for a) flat silicon surface and b) nanostructured surface with LIPSS. c) Computed reflectivity of various silicon surfaces: flat, hemicylinders of radius 125 nm, triangular with height h of 75 nm, alongside with the FDTD response of the LIPSS sample based on the AFM measurements. d)Visualization of field enhancement in silicon LIPSS modeled as parabolas with periodicity of 250 nm and height of 100 nm when irradiated with 257 nm, 550 nm, 1000, and 1500 nm light.

equations within a three-dimensional domain. The simulation first focuses on a silicon nanostructured surface situated in vacuum. The dielectric properties of silicon are modeled using the experimental values reported in [41]. For all simulations, the system is excited with an incident electromagnetic plane wave, linearly polarized, defined as $E = E_0 e^{-ikz}\hat{y}$, with $E_0 = 1$ V/m and $k = 2\pi/\lambda$ (where λ represents the wavelength). The FEM meshing refinement is set to a precision level of $\lambda/10$, and a parametric sweep is employed in the solver to comprehensively examine the behavior of the nanophotonics system.

Figure S3d shows the field enhancement achievable for LIPSS modeled as parabolic nanostructures with periodicity of 250 nm and a height of 100 nm. The nanostructures excited by a 257 nm incident field displays the largest enhancement > 3, and occurring right between the nanopatterns. For longer wavelengths, minor field enhancement is also observed, particularly for excitation above 1000 nm. However, the region where the enhancement occurs is highly localized.

4 Additional data of surface analysis using SEM and AFM

Additional SEM images of the processed sample with parameters $v = 25$ µm/s and pulse energy of $E = 27$ nJ are included in Figure S4. These were produced close to the threshold for LIPSS formation and are included here to highlight the effect of seeding into the LIPSS nanopatters by previously existing nanogrooves on the silicon surface. For example, Figure S4a shows the LIPSS nanopatterns aliging with previous grooves, while Figure S4b, c and d are magnified versions from the regions of interest including the seeding effect at the edges of the laser beam and the produced patterns in the central part of the processed area, respectively. Figure S4e shows the result of an AFM measurement performed on one of the nano-scratches that seeded the LIPSS formation. These scratches are of the order of a few tens of nanometers wide and between 10 and 20 nm deep.

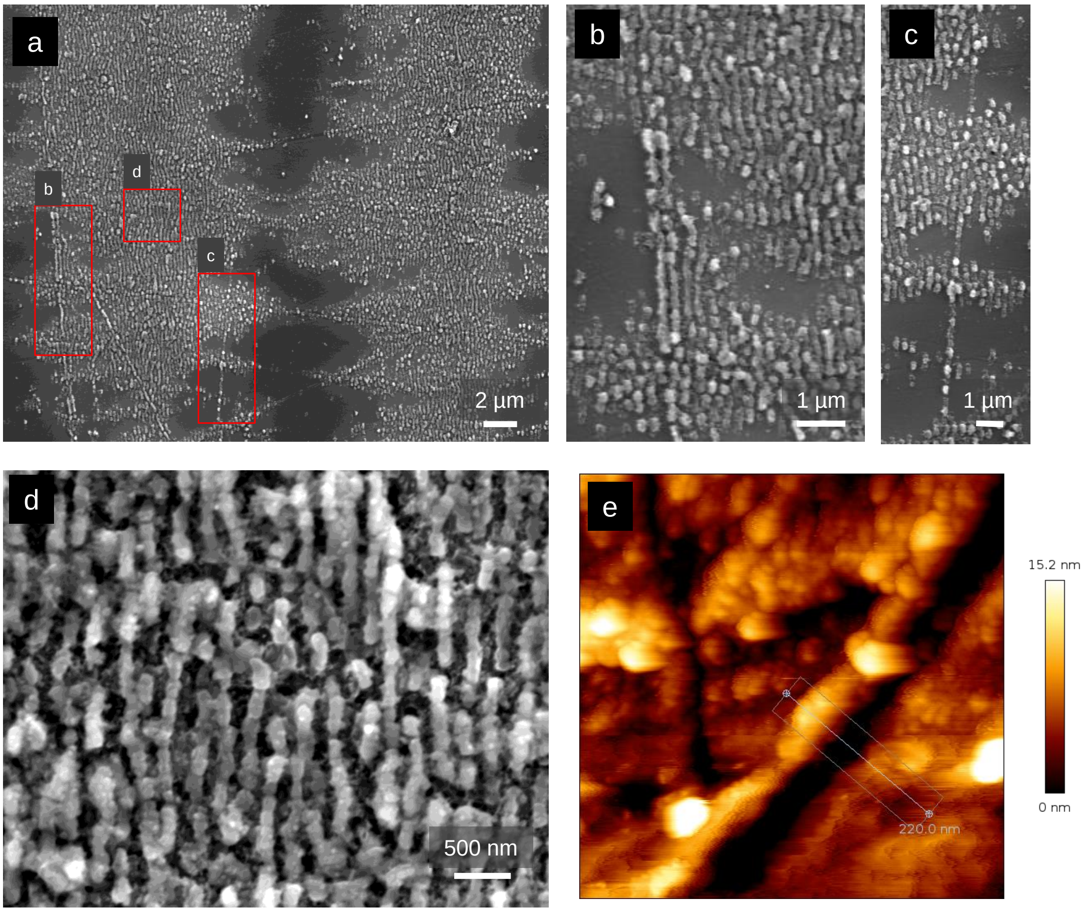

Figure S4: a) SEM image of the processed silicon sample near ablation threshold. b) c) and d) are magnified images of regions of interest including the side and central parts of the laser processed areas. e) AFM measurement of a nano-scratch that seeds the LIPSS formation.

As stated in the main text, for fluences above the ablation threshold, the formation of "worm-like" nanostructures appeared. Here we include measurements of the topography of these structures, showing that the overall peak to valley distance exceeds 800 nm while the distance between crests is approximately 1 µm.

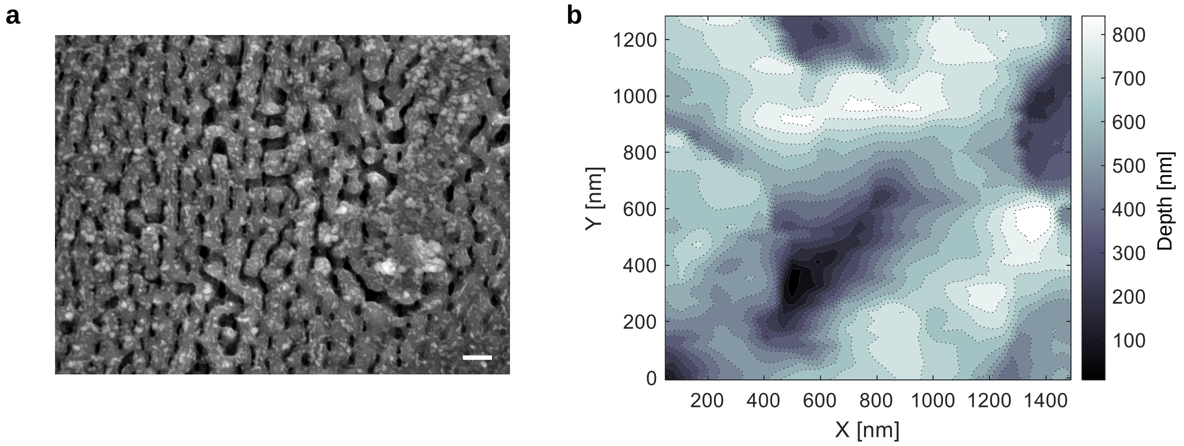

Figure S5: Surface topography of "worm-like" nanostructures as observed by SEM (a) and AFM (b).

References

[1] Tsing-Hua Her, Richard J. Finlay, Claudia Wu, Shrenik Deliwala, and Eric Mazur. Microstructuring of silicon with femtosecond laser pulses. *Applied Physics Letters*, 73(12):1673–1675, 09 1998.

[2] E. Skantzakis, V. Zorba, D.G. Papazoglou, I. Zergioti, and C. Fotakis. Ultraviolet laser microstructuring of silicon and the effect of laser pulse duration on the surface morphology. *Applied Surface Science*, 252(13):4462–4466, 2006. Proceedings of the European Materials Research society 2005 - Symposium-J: Advances in Laser and Lamp Processing of Functional Materials.

[3] V. Zorba, N. Boukos, I. Zergioti, and C. Fotakis. Ultraviolet femtosecond, picosecond and nanosecond laser microstructuring of silicon: structural and optical properties. *Appl. Opt.*, 47(11):1846–1850, Apr 2008.

[4] Godai Miyaji, Kenzo Miyazaki, Kaifeng Zhang, Takakazu Yoshifuji, and Junya Fujita. Mechanism of femtosecond-laser-induced periodic nanostructure formation on crystalline silicon surface immersed in water. *Opt. Express*, 20(14):14848–14856, Jul 2012.

[5] Feng Liang and Réal Vallée. Femtosecond laser-induced ultra-fine nanostructures on silicon surface. *Opt. Mater. Express*, 6(10):3330–3338, Oct 2016.

[6] M. G. Rahimian, A. Jain, H. Larocque, P. B. Corkum, E. Karimi, and V. R. Bhardwaj. Spatially controlled nano-structuring of silicon with femtosecond vortex pulses. *Scientific Reports*, 10(1):12643, Jul 2020.

[7] V. S. Vendamani, Syed Hamad, V. Saikiran, A. P. Pathak, S. Venugopal Rao, V. V. Ravi Kanth Kumar, and S. V. S. Nageswara Rao. Synthesis of ultra-small silicon nanoparticles by femtosecond laser ablation of porous silicon. *Journal of Materials Science*, 50(4):1666–1672, Feb 2015.

[8] A.Y. Vorobyev and Chunlei Guo. Direct creation of black silicon using femtosecond laser pulses. *Applied Surface Science*, 257(16):7291–7294, 2011.

[9] Svetoslav Koynov, Martin S. Brandt, and Martin Stutzmann. Black nonreflecting silicon surfaces for solar cells. *Applied Physics Letters*, 88(20):203107, 05 2006.

[10] J.Y.-H. Chai, B.T. Wong, and S. Juodkazis. Black-silicon-assisted photovoltaic cells for better conversion efficiencies: a review on recent research and development efforts. *Materials Today Energy*, 18:100539, 2020.

[11] Hele Savin, Päivikki Repo, Guillaume von Gastrow, Pablo Ortega, Eric Calle, Moises Garín, and Ramon Alcubilla. Black silicon solar cells with interdigitated back-contacts achieve 22.1% efficiency. *Nature Nanotechnology*, 10(7):624–628, Jul 2015.

[12] Yuya Onitsuka and Kentaro Imamura. Improvement of blue response of black si solar cells due to graded band structure. *Physica E: Low-dimensional Systems and Nanostructures*, 140:115196, 2022.

[13] Jihun Oh, Hao-Chih Yuan, and Howard M. Branz. An 18.2%-efficient black-silicon solar cell achieved through control of carrier recombination in nanostructures. *Nature Nanotechnology*, 7(11):743–748, Nov 2012.

[14] Jeff F. Young, J. S. Preston, H. M. van Driel, and J. E. Sipe. Laser-induced periodic surface structure. ii. experiments on ge, si, al, and brass. *Phys. Rev. B*, 27:1155–1172, Jan 1983.

[15] Reina Miyagawa, Daisuke Kamibayashi, Hirotaka Nakamura, Masaki Hashida, Heishun Zen, Toshihiro Somekawa, Takeshi Matsuoka, Hiroyuki Ogura, Daisuke Sagae, Yusuke Seto, Takahisa Shobu, Aki Tominaga, Osamu Eryu, and Norimasa Ozaki. Crystallinity in periodic nanostructure surface on si substrates induced by near- and mid-infrared femtosecond laser irradiation. *Scientific Reports*, 12(1):20955, Dec 2022.

[16] Shota Kawabata, Shi Bai, Kotaro Obata, Godai Miyaji, and Koji Sugioka. Two-dimensional laser-induced periodic surface structures formed on crystalline silicon by ghz burst mode femtosecond laser pulses. *International Journal of Extreme Manufacturing*, 5(1):015004, 2023.

[17] Wei Liu, Lan Jiang, Weina Han, Jie Hu, Xiaowei Li, Ji Huang, Shenghua Zhan, and Yongfeng Lu. Manipulation of lipss orientation on silicon surfaces using orthogonally polarized femtosecond laser double-pulse trains. *Opt. Express*, 27(7):9782–9793, Apr 2019.

[18] Pengjun Liu, Lan Jiang, Jie Hu, Weina Han, and Yongfeng Lu. Direct writing anisotropy on crystalline silicon surface by linearly polarized femtosecond laser. *Opt. Lett.*, 38(11):1969–1971, Jun 2013.

[19] Pengjun Liu, Lan Jiang, Jie Hu, Shuai Zhang, and Yongfeng Lu. Self-organizing microstructures orientation control in femtosecond laser patterning on silicon surface. *Opt. Express*, 22(14):16669–16675, Jul 2014.

[20] M. Barberoglou, G. D. Tsibidis, D. Gray, E. Magoulakis, C. Fotakis, E. Stratakis, and P. A. Loukakos. The influence of ultra-fast temporal energy regulation on the morphology of si surfaces through femtosecond double pulse laser irradiation. *Applied Physics A*, 113(2):273–283, Nov 2013.

[21] Jörn Bonse, Arkadi Rosenfeld, and Jörg Krüger. On the role of surface plasmon polaritons in the formation of laser-induced periodic surface structures upon irradiation of silicon by femtosecond-laser pulses. *Journal of Applied Physics*, 106(10):104910, 11 2009.

[22] Urs Zywietz, Andrey B. Evlyukhin, Carsten Reinhardt, and Boris N. Chichkov. Laser printing of silicon nanoparticles with resonant optical electric and magnetic responses. *Nature Communications*, 5(1):3402, Mar 2014.

[23] Weina Han, Lan Jiang, Xiaowei Li, Yang Liu, and Yongfeng Lu. Femtosecond laser induced tunable surface transformations on (111) Si aided by square grids diffraction. *Applied Physics Letters*, 107(25):251601, 12 2015.

[24] Thibault J.-Y. Derrien, Jörg Krüger, Tatiana E. Itina, Sandra Höhm, Arkadi Rosenfeld, and Jörn Bonse. Rippled area formed by surface plasmon polaritons upon femtosecond laser double-pulse irradiation of silicon. *Opt. Express*, 21(24):29643–29655, Dec 2013.

[25] F. Fraggelakis, E. Stratakis, and P.A. Loukakos. Control of periodic surface structures on silicon by combined temporal and polarization shaping of femtosecond laser pulses. *Applied Surface Science*, 444:154–160, 2018.

[26] R. Le Harzic, D. Dörr, D. Sauer, M. Neumeier, M. Epple, H. Zimmermann, and F. Stracke. Large-area, uniform, high-spatial-frequency ripples generated on silicon using a nanojoule-femtosecond laser at high repetition rate. *Opt. Lett.*, 36(2):229–231, Jan 2011.

[27] J. Bonse, S. Baudach, J. Krüger, W. Kautek, and M. Lenzner. Femtosecond laser ablation of silicon–modification thresholds and morphology. *Applied Physics A*, 74(1):19–25, Jan 2002.

[28] Laura Gemini, Masaki Hashida, Masahiro Shimizu, Yasuhiro Miyasaka, Shunsuke Inoue, Shigeki Tokita, Jiri Limpouch, Tomas Mocek, and Shuji Sakabe. Periodic nanostructures self-formed on silicon and silicon carbide by femtosecond laser irradiation. *Applied Physics A*, 117(1):49–54, Oct 2014.

[29] Luis Humberto Robledo-Taboada, Javier Francisco Jiménez-Jarquín, Mariela Flores-Castañeda, Antonio Méndez-Blas, Jacob Barranco-Cisneros, and Santiago Camacho-López. Single-step femtosecond laser-induced formation of coexisting microstructures in silicon. *Bulletin of Materials Science*, 46(2):92, May 2023.

[30] R. Kuladeep, L. Jyothi, Chakradhar Sahoo, D. Narayana Rao, and V. Saikiran. Optical, structural and morphological studies of nanostructures fabricated on silicon surface by femtosecond laser irradiation. *Journal of Materials Science*, 57(3):1863–1880, Jan 2022.

[31] Yulia Borodaenko, Sergey Syubaev, Stanislav Gurbatov, Alexey Zhizhchenko, Aleksey Porfirev, Svetlana Khonina, Eugeny Mitsai, Andrey V. Gerasimenko, Alexander Shevlyagin, Evgeny Modin, Saulius Juodkazis, Evgeny L. Gurevich, and Aleksandr A. Kuchmizhak. Deep subwavelength laser-induced periodic surface structures on silicon as a novel multifunctional biosensing platform. *ACS Applied Materials & Interfaces*, 13(45):54551–54560, Nov 2021.

[32] Kevin Werner, Vitaly Gruzdev, Noah Talisa, Kyle Kafka, Drake Austin, Carl M. Liebig, and Enam Chowdhury. Single-shot multi-stage damage and ablation of silicon by femtosecond mid-infrared laser pulses. *Scientific Reports*, 9(1):19993, Dec 2019.

[33] P. A. Danilov, A. A. Ionin, S. I. Kudryashov, S. V. Makarov, A. A. Rudenko, P. N. Saltuganov, L. V. Seleznev, V. I. Yurovskikh, D. A. Zayarny, and T. Apostolova. Silicon as a virtual plasmonic material: Acquisition of its transient optical constants and the ultrafast surface plasmon-polariton excitation. *Journal of Experimental and Theoretical Physics*, 120(6):946–959, Jun 2015.

[34] Sandra Höhm, Marcel Herzlieb, Arkadi Rosenfeld, Jörg Krüger, and Jörn Bonse. Femtosecond laser-induced periodic surface structures on silicon upon polarization controlled two-color double-pulse irradiation. *Opt. Express*, 23(1):61–71, Jan 2015.

[35] Yulia Borodaenko, Sergey Syubaev, Evgeniia Khairullina, Ilya Tumkin, Stanislav Gurbatov, Aleksandr Mironenko, Eugeny Mitsai, Alexey Zhizhchenko, Evgeny Modin, Evgeny L. Gurevich, and Aleksandr A. Kuchmizhak. On-demand plasmon nanoparticle-embedded laser-induced periodic surface structures (lipsss) on silicon for optical nanosensing. *Advanced Optical Materials*, 10(21):2201094, 2022.

[36] Sebastien Durbach and Norbert Hampp. Scan direction of circularly polarized laser beam determines the orientation of laser-induced periodic surface structures (LIPSSs) on silicon. *Applied Physics Letters*, 121(25):251601, 12 2022.

[37] Jörn Bonse and Stephan Gräf. Maxwell meets marangoni—a review of theories on laser-induced periodic surface structures. *Laser & Photonics Reviews*, 14(10):2000215, 2020.

[38] Min Jin Kang, Tae Sang Park, Minyeong Kim, Eui Sun Hwang, Seung Hwan Kim, Sung Tae Shin, and Byoung-Ho Cheong. Periodic surface texturing of amorphous-si thin film irradiated by uv nanosecond laser. *Opt. Mater. Express*, 9(11):4247–4255, Nov 2019.

[39] A. J. Pedraza, J. D. Fowlkes, and Y.-F. Guan. Surface nanostructuring of silicon. *Applied Physics A*, 77(2):277–284, Jul 2003.

[40] Raul Zazo, Javier Solis, José A. Sanchez-Gil, Rocio Ariza, Rosalia Serna, and Jan Siegel. Deep uv laser induced periodic surface structures on silicon formed by self-organization of nanoparticles. *Applied Surface Science*, 520:146307, 2020.

[41] D. E. Aspnes and A. A. Studna. Dielectric functions and optical parameters of si, ge, gap, gaas, gasb, inp, inas, and insb from 1.5 to 6.0 ev. *Phys. Rev. B*, 27:985–1009, Jan 1983.
